# Exploring the Potential Role of Oligodendrocyte-Associated PIP4K2A in Alzheimer’s Disease Complicated with Type 2 Diabetes Mellitus via Multi-Omic Analysis

**DOI:** 10.3390/ijms25126640

**Published:** 2024-06-17

**Authors:** Doan Phuong Quy Nguyen, Amadou Wurry Jallow, Yi-Fang Lin, Yung-Feng Lin

**Affiliations:** 1 Ph.D. Program in Medical Biotechnology, College of Medical Science and Technology, Taipei Medical University, New Taipei City 235, Taiwan; ndpquy@hueuni.edu.vn (D.P.Q.N.); d609111003@tmu.edu.tw (A.W.J.); 2 Institute of Biomedicine, Hue University of Medicine and Pharmacy, Hue University, Hue City 49120, Vietnam; 3 Department of Medical Genetics, Hue University of Medicine and Pharmacy, Hue University, Hue City 49120, Vietnam; 4 Department of Laboratory Medicine, Taipei Medical University—Shuang Ho Hospital, New Taipei City 235, Taiwan; 08310@s.tmu.edu.tw; 5 School of Medical Laboratory Science and Biotechnology, College of Medical Science and Technology, Taipei Medical University, New Taipei City 235, Taiwan; 6 Department of Laboratory Medicine, Taipei Medical University Hospital, Taipei City 110, Taiwan

**Keywords:** Alzheimer’s disease, bioinformatics, biomarker, PIP4K2A, single-cell sequencing, type 2 diabetes mellitus

## Abstract

Alzheimer’s disease (AD) and type 2 diabetes mellitus (T2DM) are two common diseases that affect the elderly population worldwide. The identification of common genes associated with AD and T2DM holds promise for potential biomarkers and intriguing pathogenesis of these two complicated diseases. This study utilized a comprehensive approach by integrating transcriptome data from multiple cohorts, encompassing both AD and T2DM. The analysis incorporated various data types, including blood and tissue samples as well as single-cell datasets, allowing for a detailed assessment of gene expression patterns. From the brain region-specific single-cell analysis, *PIP4K2A*, which encodes phosphatidylinositol-5-phosphate 4-kinase type 2 alpha, was found to be expressed mainly in oligodendrocytes compared to other cell types. Elevated levels of *PIP4K2A* in AD and T2DM patients’ blood were found to be associated with key cellular processes such as vesicle-mediated transport, negative regulation of autophagosome assembly, and cytosolic transport. The identification of *PIP4K2A*’s potential roles in the cellular processes of AD and T2DM offers valuable insights into the development of biomarkers for diagnosis and therapy, especially in the complication of these two diseases.

## 1. Introduction

Alzheimer’s disease (AD) is a prevalent neurodegenerative disorder that primarily affects the elderly population worldwide. It is characterized by a progressive decline in memory and thinking skills, eventually leading to the inability to carry out the simplest tasks [1]. AD accounts for 60–70% of dementia cases among older adults worldwide. In 2022, an estimated 6.7 million older Americans were living with AD, leading to over 121,499 deaths and unpaid dementia caregiving valued at approximately USD 339.5 billion [2]. The diminished ability of individuals with AD to function independently and care for themselves results in a lower quality of life for both patients and their caregivers. This situation creates significant economic and time burdens for families and society [3]. At present, there is no effective cure or clinically approved biomarker for the detection of AD pathogenesis. AD can only be precisely diagnosed after the excessive formation of amyloid-beta (Aβ) plaque and neurofibrillary tangle in the brain, which requires the use of positron emission tomography scans or cerebrospinal fluid examination [4,5]. These methods are costly and invasive, making them impractical for large-scale screening. Thus, less interfering methods for AD diagnosis, which could improve early diagnosis and prognosis, are needed.

Investigations have uncovered that the causes of AD are multifaceted, encompassing both environmental influences and genetic predispositions [1,6]. It was hypothesized that impaired metabolic function plays a role in the development of degenerative diseases [7,8]. Specifically, there is a correlation between the progression of neurodegenerative disorders and metabolic disorders [9]. In contemporary understanding, type 1 and type 2 diabetes mellitus (DM) have garnered considerable recognition. Metabolic disturbance including hyperglycemia and dyslipidemia in DM leads to the formation of advanced glycation end products and aldehydic adducts, which are the major contributors to insulin resistance [10,11,12]. Type 2 diabetes mellitus (T2DM) is recognized as a risk factor for dementia due to its characteristic peripheral insulin resistance [13]. Notably, the transcriptomic alterations in cortical neurons and associated neurovascular unit cells in the ageing brain with T2DM are well-documented, revealing significant changes in insulin signaling pathways, cell cycle regulation, and inflammatory mediators, which may contribute to neuronal damage and dysfunction associated with diabetic dementia. In addition, insulin activity is related to the key proteins affected in neurodegenerative conditions, such as the amyloid precursor protein (APP) and its vesicle-transporting machinery in the central nervous system [14,15]. A meta-analysis also provides evidence that the development of AD is increased in elderly individuals with T2DM [16]. The role of diabetes at different stages of AD was also highlighted through transcriptomic and network analyses, which have unveiled shared and unique pathways and potential therapeutic targets. These studies have shown that T2DM may influence the progression of AD through various molecular pathways, including inflammation, infectious diseases, and the phosphoinositide 3-kinases/protein kinase (PI3K/AKT) signaling pathway [17]. Furthermore, identifying comorbidity genes shared between AD and T2DM has provided valuable insights into the common molecular mechanisms contributing to the memory and cognitive impairments observed in both diseases. Network clustering based on protein–protein interaction networks has identified gene clusters with significant impacts on memory and cognition, offering potential biomarkers and therapeutic targets [18]. Nonetheless, the exact mechanisms underlying the link between AD and T2DM are still not fully understood and are a topic of ongoing research.

In this context, our study aims to explore the central genes involved in both AD and T2DM and the common underlying pathways between these disorders. We employed a comprehensive approach by integrating transcriptome data from diverse cohorts including AD and T2DM. We analyzed datasets from AD patients’ blood and tissue samples, as well as T2DM blood samples, to identify hub genes that may serve as crucial molecular targets and demonstrate consistent expression patterns across these two diseases through transcriptional profiling. Additionally, we conducted enrichment analysis to understand the molecular functions of common genes and evaluated their predictive potential using area under the curve (AUC) scores in a machine learning approach. Furthermore, single-cell datasets from different brain regions (superior frontal gyrus, entorhinal cortex, and hippocampus region) were analyzed to gain a more detailed and reliable assessment of gene expression patterns. We investigated the specific role of phosphatidylinositol-5-phosphate 4-kinase type 2 alpha (*PIP4K2A*) in oligodendrocyte cell type, which is significantly associated with vesicle-mediated transport, negative regulation of autophagosome assembly, and cytosolic transport pathways. These findings imply a potential involvement of *PIP4K2A* in cellular processes associated with AD and T2DM, suggesting its possible contribution to the pathogenesis of these two disorders.

## 2. Results

### 2.1. Transcriptional Profiling of Hub Genes in AD and T2DM

**Figure 1:**
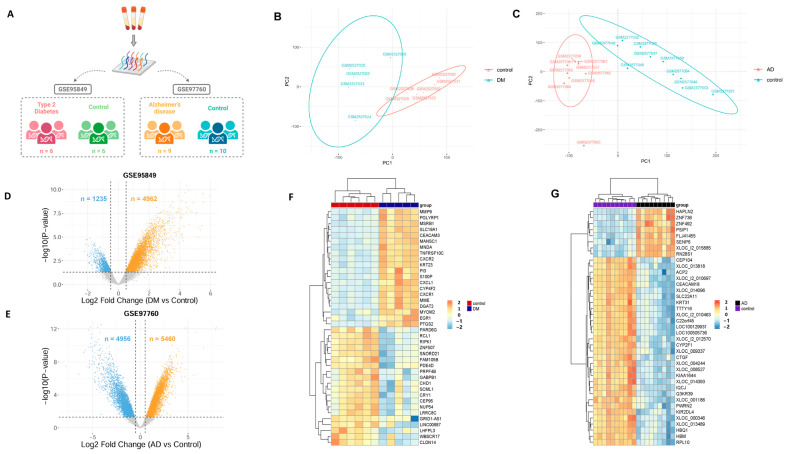
The expression patterns observed in the microarray dataset provide distinctive characteristics associated with AD and T2DM. (**A**) Summary of AD and T2DM microarray dataset. (**B**) The principal component analysis map of GSE95849 (T2DM dataset) in two phenotype samples. (**C**) The principal component analysis (PCA) map of GSE97760 (AD dataset) in two phenotype samples. (**D**,**E**) The volcano plot illustrates two distinct lists of differential gene expressions (DEGs) in T2DM and AD compared to healthy control samples, respectively. The volcano plot depicts upregulated genes as orange dots, while downregulated genes are represented by blue dots. Non-significant genes are shown as gray dots. The thresholds used for defining differential expression were set as *p*-value < 0.05 and |log2FC| > 0.5. (**F**,**G**) The heatmap displays the top significant genes that are differentially expressed in the T2DM, and AD groups compared to the control samples. The gene expression matrix was scaled to a range of (−2, 2), with corresponding colors ranging from blue to red.

Bulk transcriptomic data from blood samples of nine AD cases and ten controls, as well as six T2DM samples and six controls (Figure 1A), were collected. Subsequently, we employed a five-step multi-disciplinary approach for gene prioritization, aiming to select disease-relevant genes. In the initial step, we used principal component analysis to identify and exclude potential outliers within the datasets. Outliers were determined based on their proximity to the coordinate system, specifically focusing on samples that exhibited similar positions between the disease and control groups, as well as samples that deviated significantly from the rest. These deviations could arise from various factors, such as inconsistencies in sample collection procedures or other sources of variability (Figure 1B,C). In the T2DM dataset, we identified “GSM2527027” as an outlier based on its deviation from the expected pattern observed in the other samples. Consequently, we made the decision to exclude this sample from the original dataset to ensure the accuracy and robustness of our subsequent analyses (Supplementary Figure S1). However, it is worth noting that no outliers were detected in the AD dataset, indicating a higher degree of conformity and consistency among the samples in this dataset.

**Figure 2:**
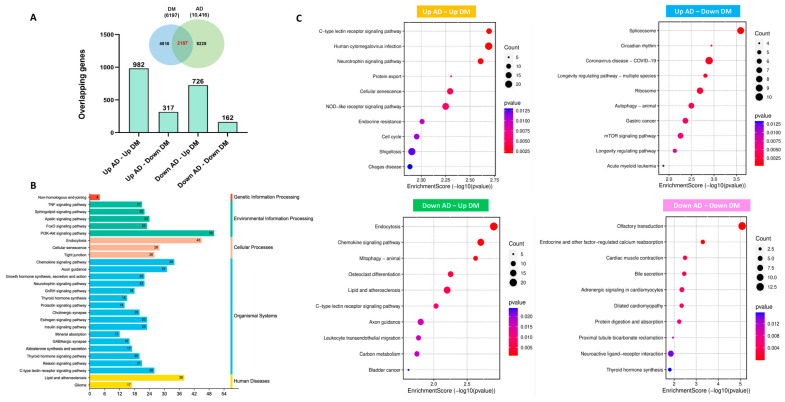
The overlapping DEGs of AD and T2DM. (**A**) The overlap between the DEG lists of AD and T2DM resulted in a total of 2187 DEGs. These DEGs were further categorized into four sub-gene sets: Up AD–Up DM, Up AD–Down DM, Down AD–Up DM, and Down AD–Down DM. (**B**) The Kyoto Encyclopedia of Genes and Genomes (KEGG) enrichment analysis was performed using the 2187 DEGs as input. The results of the analysis were visualized in a bar chart, which represents the KEGG annotation categories. (**C**) The distinct enrichment pathways for each gene set in (**A**) were identified through KEGG analysis. The pathway analysis was conducted using the clusterProfiler package, and pathways with a significance threshold of *p*-value < 0.05 were considered significant. The resulting pathways were visualized in a dot plot.

Next, we conducted the differential gene expression (DGE) analysis between AD and control as well as T2DM and control samples using two gene expression omnibus (GEO) datasets. In the GSE97760 dataset, we identified a total of 10,416 differentially expressed genes (DEGs) that met the pre-defined threshold of *p*-value < 0.05 and |log2FC| > 0.5. Similarly, in the GSE95849 dataset, we found 6197 DEGs that satisfied the same threshold criteria (Figure 1D,E). We then performed an analysis to identify overlapping genes between the DEGs of AD and T2DM. This analysis resulted in a final set of 2187 genes, which were further categorized into four distinct subsets based on their expression patterns: upregulated in both AD and T2DM (982 genes), upregulated in AD and downregulated in T2DM (317 genes), downregulated in AD and upregulated in T2DM (726 genes), and downregulated in both AD and T2DM (162 genes) (Figure 2A). Subsequently, a Kyoto Encyclopedia of Genes and Genomes (KEGG) enrichment analysis was conducted using the 2187 DEGs as input, and the results were presented in a bar chart to represent the KEGG annotation categories (Figure 2B). Additionally, the distinct enrichment pathways for each gene set in Figure 2A were identified through KEGG analysis using the clusterProfiler package (version 4.10.1), considering pathways with a significance threshold of *p*-value < 0.05 as significant (Figure 2C).

### 2.2. Protein–Protein Interaction Network Uncovered Distinct Biological Patterns

**Figure 3:**
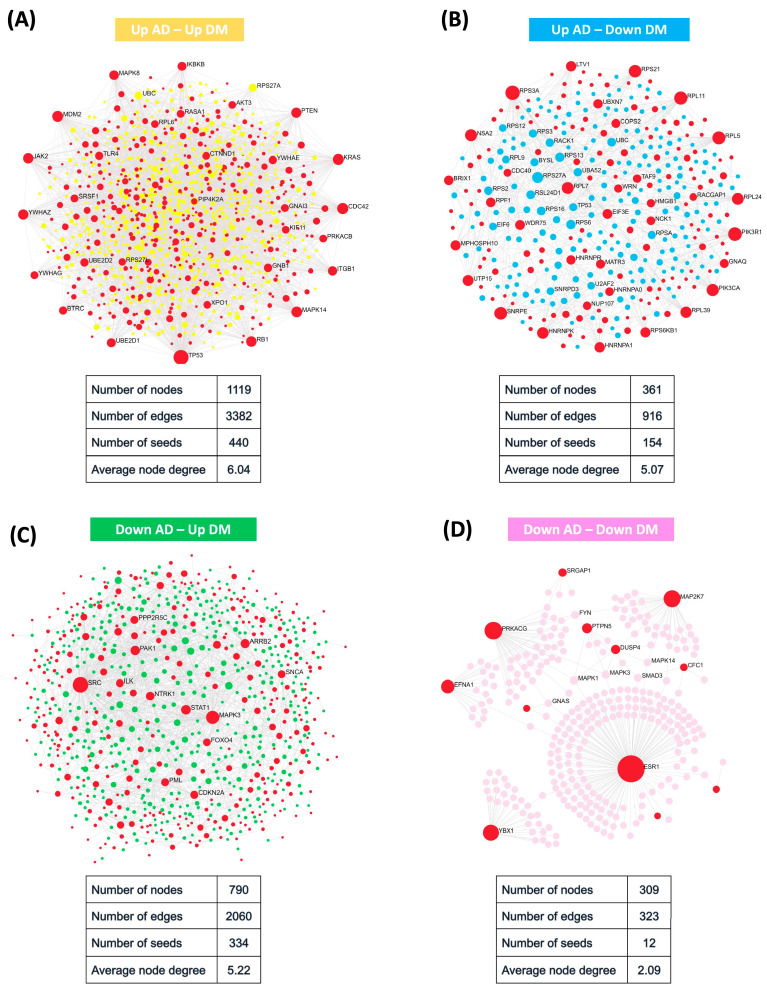
Protein–protein interaction networks. (**A**) Network of “Up AD–Up DM” gene set. (**B**) Network of “Up AD–Down DM” gene set. (**C**) Network of “Down AD–Up DM” gene set. (**D**) Network of “Down AD–Down DM” gene set. In each network, the gene sets were used as input, with the “seed” genes represented in red color. Significant proteins that correspond to the seed proteins were depicted as seed nodes and color-coded accordingly: yellow for ‘Up AD–Up DM’, blue for ‘Up AD–Down DM’, green for ‘Down AD–Up DM’, and pink for ‘Down AD–Down DM’. The transparency gradient of the nodes is based on the node degree.

DEGs alone may not necessarily indicate their direct significance to the phenotype under study. Instead, it is possible that the observed differential expression reflects their indirect association within a larger gene network. To construct the protein–protein interaction (PPI) networks for each gene set, we utilized the Network Analyst tool (https://www.networkanalyst.ca/; accessed on 24 December 2023) and selected the “STRING” database. A confidence score cutoff of 900 was applied, along with the inclusion of experimental evidence. In each network, the input genes were designated as “seeds” and significant proteins with corresponding “edges” were connected to their respective seed nodes. Specifically, for the “Up AD–Up DM” gene set, we identified 440 seed proteins from the initial set of 982 input genes. The differential expression analysis revealed interesting patterns within the gene sets associated with AD and T2DM comorbidity. The “Up AD–Up DM” gene set exhibited a substantial number of seed proteins, indicating strong network connectivity and potential functional relevance. This suggests that the upregulated genes in both AD and T2DM may participate in common pathways or biological processes contributing to comorbidity. In the case of the “Down AD–Up DM” gene set, 334 seed proteins were found among the 726 input genes. Similarly, the “Up AD–Down DM” gene set yielded 154 seed proteins from the 317 input genes. In contrast, we observed only 12 genes in the “Down AD–Down DM” gene set (Figure 3).

### 2.3. Identification of AD-Predictive Significance Genes across Multiple Datasets

Along with the blood dataset (GSE97760), we obtained additional microarray datasets from various brain regions implicated in the development of AD, including the superior frontal gyrus (GSE48350), the entorhinal cortex and the hippocampus (GSE5281). These brain regions are well-known sources of AD. Using the 2187 DEGs identified in our previous analysis as input, we calculated the area under the curve (AUC) score for each gene in classifying patients as AD or normal controls within the GSE97760 dataset. We then validated the AUC scores of these genes in each individual brain region dataset and selected genes that had an AUC score equal to or higher than 0.7.

**Figure 4:**
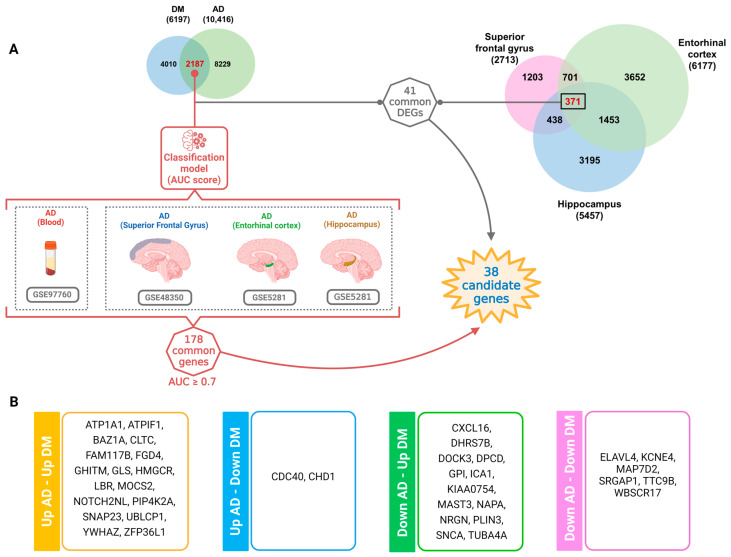
Identification of AD-predictive significance genes across multiple datasets. (**A**) Workflow summarizing the identification of AD-predictive significance genes across multiple datasets. (**B**) Characterization of the 38 genes in the final set, categorized into the four gene sets mentioned above, highlighting their respective characteristics.

We identified a set of 178 genes that met the AUC threshold criteria, including blood samples and three distinct brain region tissues. Furthermore, we performed a comparison between the tissue common DEG list (317 genes) and 2187 common DEGs from AD and T2DM blood datasets to determine if the DEGs in blood samples exhibited similar characteristics in tissue samples. This analysis revealed 41 common DEGs, which were further compared to the 178 genes with an AUC score ≥ 0.7. As a result, we identified a total of 38 candidate genes that represent a novel signature potentially involved in the common pathogenesis of AD and T2DM (Figure 4A). Interestingly, among these 38 potential genes, we observed distinct patterns of gene expression changes. Specifically, 17 genes were identified within the “Up AD–Up DM” gene set, indicating an upregulation in both AD and T2DM conditions. Only two genes were found in the “Up AD–Down DM” gene set. Additionally, 13 genes were classified in the “Down AD–Up DM”. Finally, six genes were assigned to the “Down AD–Down DM” gene set (Figure 4B).

Our next objective was to identify genes that displayed consistent expression patterns between disease samples (AD and T2DM) and control samples through differential gene expression analysis. We established criteria whereby genes that were upregulated in T2DM (in blood samples) were expected to exhibit upregulation in AD (in both blood samples and the three brain region tissue samples), and vice versa. Following our analysis, we identified eight genes out of the initial set of 38 genes that met our criteria (Table 1). Specifically, *BAZ1A*, *FGD4*, *NOTCH2NL1*, *PIP4K2A*, *SNAP23*, and *ZFP36L1* were found to be consistently upregulated in all five datasets. Conversely, *ELAVL4* and *MAP7D2* exhibited consistent downregulation in these datasets. These eight genes will be retained for further analysis in subsequent steps.

### 2.4. Transcriptional Diversity in AD and Control Samples Revealed by Single-Cell Human Brain Region Atlas

**Figure 5:**
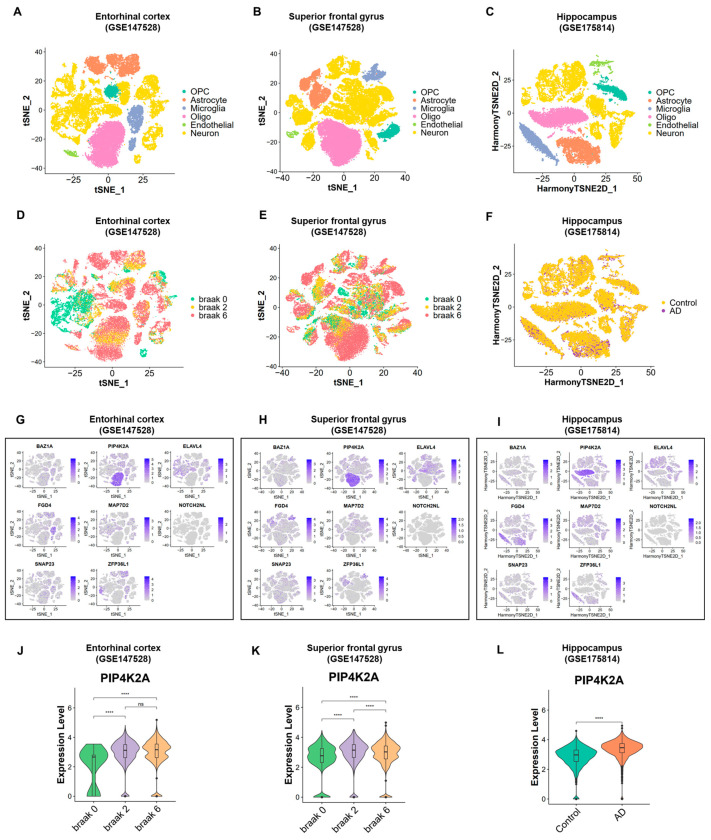
Transcriptional diversity in AD and control samples revealed by single-cell Human Brain Region Atlas. (**A**–**C**) t-distributed Stochastic Neighbor Embedding (t-SNE) visualization of three different single-cell datasets obtained from the superior frontal gyrus (GSE147528), entorhinal cortex (GSE147528), and hippocampus (GSE175814). (**D**–**F**) Corresponding t-SNEs of the brain regions depict the cellular distribution in different conditions, including Braak 0, Braak 2, and Braak 6 stages (superior frontal gyrus and entorhinal cortex datasets), as well as AD and control states (hippocampus dataset). (**G**–**I**) Gene expression patterns of 8 selected genes plotted on the t-SNE coordinates shown in (**A**), PIP4K2A displayed specific expression in oligodendrocytes. (**J**–**L**) Violin plots depicting the significant differences in gene expression levels of PIP4K2A between AD and control samples (hippocampus), between early and advanced Braak stages (entorhinal cortex and superior frontal gyrus). ns: not significant; **** *p* < 0.0001.

To strengthen our findings and increase the reliability of the results obtained from microarray data, we conducted additional analysis using single-cell data from corresponding brain regions. Specifically, we analyzed single-cell datasets from the superior frontal gyrus (GSE147528), entorhinal cortex (GSE147528), and hippocampus (GSE175814), aiming to maintain consistency with the tissue types investigated in the microarray data. The preprocessing steps for the single-cell data followed the methodologies described in the original studies, including filtering and quality control, data normalization, dimensionality reduction, clustering, and t-distributed Stochastic Neighbor Embedding (t-SNE) visualization (Figure 5A–C). All three single-cell datasets exhibited similar cell type annotations, with cells clustered into Oligodendrocyte, Oligodendrocyte progenitor cell, Microglia, Astrocyte, Endothelial, and Neuron. Corresponding t-SNEs of the brain regions illustrate the cellular distribution across distinct conditions, encompassing Braak 0, Braak 2, and Braak 6 stages (from superior frontal gyrus and entorhinal cortex datasets), along with AD and control states (from hippocampus dataset) (Figure 5D–F).

Next, we proceeded to examine the expression levels of each gene from the list of eight genes obtained through the above result (Figure 5G–I). Our selection criteria were as follows: for genes that were significantly upregulated in AD samples compared to control samples in the microarray data, we expected to observe a similar trend in the single-cell analysis when comparing AD samples to control samples, particularly within specific cell types. Similarly, for genes that were downregulated in AD samples compared to control samples in the microarray data, we anticipated a consistent trend in the single-cell analysis.

Overall, we identified one gene, PIP4K2A, that met all the specified criteria. Firstly, PIP4K2A showed significant enrichment in oligodendrocytes across the entorhinal cortex, prefrontal cortex, and hippocampus regions. This gene was initially upregulated in AD compared to control blood samples, and we observed a significant increase in this gene expression level in Braak stage 2 and Braak stage 6 from the entorhinal cortex and superior frontal gyrus dataset, as well as in AD samples compared to control samples from the hippocampus dataset (Figure 5J–L). The specific expression level of PIP4K2A in comparison to oligodendrocytes and other cell types is described in Supplementary Figure S2.

### 2.5. Exploration of PIP4K2A Expression Level in Multi-Cohorts and Its Key Biological Pathways

**Figure 6:**
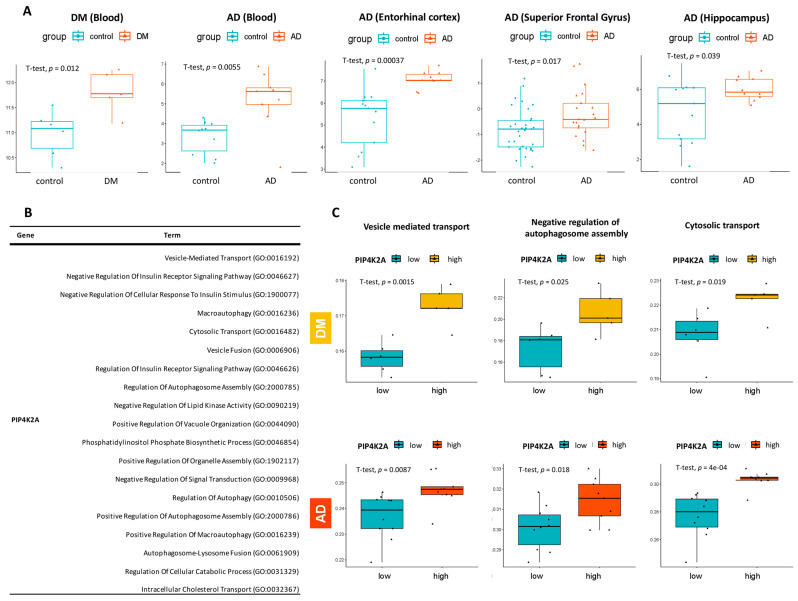
Comprehensive characterization of PIP4K2A across multiple brain region microarray datasets. (**A**) Boxplot illustrating the expression level distribution of PIP4K2A in the diseases and control groups, including T2DM blood samples, AD blood samples, and three different brain region tissue samples. The *p*-value is calculated using a *t*-test. (**B**) The table presents the contribution of PIP4K2A in multiple biological process pathways, obtained from the Gene Ontology 2023 database. (**C**) The high expression of PIP4K2A is associated with the upregulated levels of biological processes such as “vesicle-mediated transport”, “negative regulation of autophagosome assembly”, and “cytosolic transport” in both T2DM and AD datasets.

To confirm the differential expression of PIP4K2A observed in the DGE analysis, we performed independent *t*-tests to compare the expression levels of this gene between the disease (AD or T2DM) and control groups. Our results consistently demonstrated significant upregulation of PIP4K2A in the disease cohorts compared to the control cohorts, both in blood samples and the three different brain region datasets (Figure 6A).

Considering the potential role of PIP4K2A in the comorbidity between T2DM and AD, we examined its involvement in biological pathways associated with both conditions. We conducted a comprehensive analysis using the Gene Ontology-Biological Process 2023 database and filtered the pathways based on their biological relevance to AD and T2DM, guided by a thorough literature review. As a result, we identified 19 potential pathways that are depicted in Figure 6B. Next, we assessed the concordance of pathway scores across distinct phenotypic states, specifically comparing the high-expression group and the low-expression group of PIP4K2A. The high expression of PIP4K2A is consistently associated with the upregulated levels of key biological processes involved in cellular transport and regulation. Specifically, our analysis reveals that PIP4K2A is involved in “vesicle-mediated transport”, “negative regulation of autophagosome assembly” and “cytosolic transport”. Moreover, regarding the immune infiltration, we observed that the elevated expressions of PIP4K2A in T2DM and AD were significantly associated with the B lineage MCP counter (Supplementary Figure S3).

### 2.6. Validation of PIP4K2A Encoded Protein Level through Western Blotting

**Figure 7:**
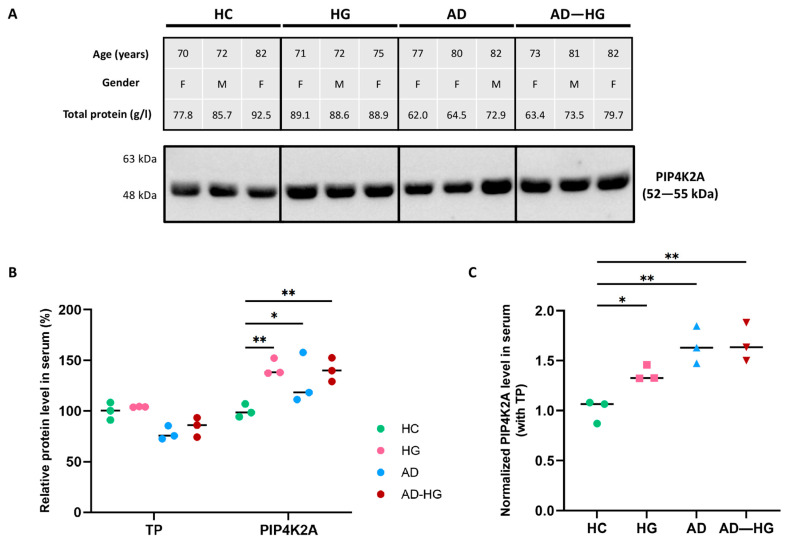
Western blot analysis of PIP4K2A in human serum. (**A**) PIP4K2A level in serum samples from healthy control (HC), hyperglycemia (HG), AD, and AD with hyperglycemia (AD–HG) patients via Western blotting. The study included two females and one male in each group. (**B**) The band densities of PIP4K2A and the total protein (TP) levels were normalized against the average values of the HC samples. (**C**) Normalized PIP4K2A levels in serum against the TP values within the same sample group; * *p* < 0.05; ** *p* < 0.01.

To validate the multi-omic findings, we performed Western blot experiments to evaluate the relative level of PIP4K2A in the human serum samples from four distinct groups: healthy controls (HC), individuals with hyperglycemia (HG), AD patients (AD), and patients with both AD and hyperglycemia (AD–HG) (Figure 7A). Significant increases in PIP4K2A levels were found in the serum samples of individuals from the HG, AD, and AD–HG groups compared to those of healthy controls, which was demonstrated not only in direct measurements (Figure 7B) but also in total protein-normalized values (Figure 7C).

## 3. Discussion

AD and T2DM are two complex and prevalent disorders with significant impacts on global health that pose major challenges for health care systems. Previous studies have demonstrated a significant link between AD and T2DM, suggesting that they may involve some common molecular and cellular pathways [19,20,21]. Furthermore, emerging evidence suggests that T2DM may increase the risk of developing AD [22,23]. This emphasizes the importance of researching the interactions between these two disorders. Investigating the intertwining pathogenesis of AD and T2DM could provide new insights into the etiology and outcomes of both diseases, as well as new avenues for diagnosis, prevention, and treatment.

In this study, the set of 2187 overlapping DEGs from AD and T2DM datasets was used to calculate the AUC score for each gene in classifying patients as AD, T2DM or normal controls within the blood and tissue data collections. By validating the AUC scores of these common DEGs across different types of datasets, we enhanced the reliability and generalizability of our findings, thereby identifying genes that hold promise as potential biomarkers or therapeutic targets in AD research. Subsequently, our investigations delved into brain tissue samples from the superior frontal gyrus, entorhinal cortex, and hippocampus. These regions are profoundly affected in AD, rendering them essential for the study of molecular alterations linked to this disorder [24,25,26]. Through our AUC-based sorting, we identified 38 common genes and selected eight candidates that met our criteria: *BAZ1A*, *ELAVL4*, *FGD4*, *MAP7D2*, *NOTCH2NL*, *PIP4K2A*, *SNAP23*, and *ZFP36L1*. We expected these genes to show consistent expression changes in AD samples compared to controls in both blood and tissue microarray datasets, as well as in blood data from DM patients versus healthy individuals. Additionally, we anticipated finding a similar pattern in single-cell profiling that would align with the gene expression changes observed in AD microarray datasets. Among the eight candidate genes, *PIP4K2A* demonstrated a specific expression in oligodendrocytes and a significant increase in AD patients compared to controls in single-cell datasets from the superior frontal gyrus, entorhinal cortex, and hippocampus. Elevated *PIP4K2A* levels in both AD and diabetic patients’ blood are possibly associated with vesicle-mediated transport, negative regulation of autophagosome assembly, and cytosolic transport. Considering the gene’s molecular functions and the protein it encodes, we evaluated the expression levels of PIP4K2A in serum samples obtained from patients with hyperglycemia, AD, and those with both AD and hyperglycemia, in comparison to samples from healthy individuals using Western blotting. As anticipated, the analysis indeed verified a considerable increase in PIP4K2A levels across all patient groups, encompassing those with hyperglycemia, AD, and individuals with both AD and hyperglycemia, when compared with healthy controls (Figure 7).

The presence of PIP4K2A in the blood, despite its predominant expression in oligodendrocytes, can be attributed to several possible factors. Firstly, oligodendrocytes, like many cell types, release exosomes and other extracellular vesicles that can contain a variety of proteins [27]. These vesicles can enter the bloodstream crossing the blood–brain barrier (BBB) and contribute to the detectable levels of proteins such as PIP4K2A in the blood [28]. Additionally, defective insulin signaling and mitochondrial dysfunction, which lead to tissue damage and neuroinflammation in conditions such as AD and T2DM, can cause the release of intracellular contents from the brain into the blood [29]. The inflammation and oxidative stress characteristics of both diseases can impair the BBB, allowing more brain-derived proteins to enter circulation [30]. These combined factors may explain the detection of PIP4K2A in the blood despite its primary expression in oligodendrocytes.

PIP4K2A, also known as phosphatidylinositol-5-phosphate 4-kinase (PI5P4K)-α, is a member of the PI5P4Ks family, consisting of three isoforms (α, β, and γ) encoded by *PIP4K2A*, *PIP4K2B*, and *PIP4K2C*, respectively [31]. Among these isoforms, PIP4K2A exhibits the highest level of activity [32]. Its molecular functions are primarily associated with its role as a kinase enzyme involved in phosphoinositide metabolism. By phosphorylating PI(5)P, PIP4K2A catalyzes the production of PI(4,5)P2, a critical phosphoinositide that regulates various cellular processes including intracellular signaling, membrane trafficking, cytoskeletal organization, ion channel regulation, and cell proliferation [33,34,35]. PIP4K2A is a multifunctional enzyme that plays crucial roles in both diabetes and AD through its regulation of PI(4,5)P2 levels. By influencing cell signaling, growth, and function, PIP4K2A contributes to the pathogenesis of these diseases. PIP4K2A is crucial for maintaining PI(4,5)P2 levels, which regulate several cellular signaling pathways. PI(4,5)P2 serves as a substrate for PI3Ks and phospholipase C, influencing downstream signaling cascades that affect cell growth, survival, and proliferation. Additionally, PI(4,5)P2 is vital for actin cytoskeleton dynamics, impacting cell shape, motility, and adhesion [36].

PIP4K2A is integral to lipid metabolism and PI3K/Akt signaling, both of which are crucial in the pathophysiology of AD and T2DM [37]. PIP4K2A’s role extends beyond its kinase activity in phosphoinositide metabolism; it also influences the PI3K/Akt pathway, a central conduit for cellular growth, survival, and metabolic regulation. In AD, aberrant lipid metabolism and disrupted Akt signaling contribute to synaptic dysfunction and neuronal loss [38]. The Akt signaling pathway, modulated by PIP4K2A, could affect Aβ production, a key event in AD pathology [39,40]. Lipid metabolism-related genes, play a role in the progression of AD by affecting the brain’s lipid profile and influencing the development of AD pathology [41]. Cortical lipid metabolic pathway alterations were associated with early stages of AD, and these changes are linked to the dysregulation of pathways such as the peroxisome proliferator-activated receptor (PPAR) signaling pathway, glycerophospholipid metabolism, and fatty acid biosynthesis and degradation [13].

In T2DM, PIP4K2A’s involvement in lipid metabolism intersects with insulin resistance. A study has shown that PIP4Ks, including PIP4K2A, can suppress insulin signaling through a mechanism that is independent of their catalytic activity [35]. Proper PI(4,5)P2 levels are necessary for the insulin receptor and its downstream effectors to function correctly. Dysregulated PIP4K2A activity can impair insulin signaling, leading to reduced glucose uptake and metabolism, contributing to insulin resistance. PIP4K2A also maintains glucose homeostasis, as alterations in PI(4,5)P2 can disrupt glucose production and utilization, leading to hyperglycemia. Furthermore, PIP4K2A is critical for the health and function of pancreatic beta cells, whose dysfunction due to aberrant PIP4K2A activity can exacerbate diabetes progression [36,42]. Insulin resistance is also known as the linking mechanism between T2DM and AD, research on the role of insulin receptor substrate-1 modulating the PI3K/Akt insulin signaling pathway indicates that it potentiates the formation of Aβ plaques by reducing the degradation and clearance of Aβ, leading to enhanced production of Aβ and hyperphosphorylated tau in the brain with AD [43].

In AD, PIP4K2A is significant for neuronal survival and function. PI(4,5)P2 is integral to synaptic vesicle trafficking and neurotransmitter release, essential for synaptic function and plasticity. Disruptions in PIP4K2A activity can impair synaptic function, contributing to cognitive decline in AD. PIP4K2A may also influence APP processing, crucial in AD pathogenesis. Abnormal APP processing leads to Aβ peptide production, which forms plaques characteristic of AD. Altered PIP4K2A activity could affect APP processing and Aβ accumulation [36,44,45]. Understanding PIP4K2A’s roles in diabetes and AD through its regulation of PI(4,5)P2 levels highlights its potential as a therapeutic target. Targeting PIP4K2A activity may offer new strategies for managing and treating these diseases. Further research is necessary to elucidate the molecular pathways involved and develop targeted therapies to modulate PIP4K2A activity effectively.

While the direct involvement of PI5P4Kα in autophagy is still under investigation, its connections to phosphoinositide signaling and membrane dynamics suggest potential roles in autophagy processes. Autophagy is a lysosome-dependent catabolic pathway that degrades normal or dysfunctional cellular components [46]. Dysfunction of autophagy is strongly linked to a broad range of human pathophysiological conditions, such as cancers and neurodegenerative diseases, due to its critical role in cellular homeostasis maintenance, and adaptation to environmental and cellular stresses [47,48]. In a study by Laura Caberlotto et al., the essential involvement of the autophagic pathway in the pathophysiology of AD and T2DM was demonstrated [49]. Dysregulation of autophagy leads to the buildup of misfolded proteins and neurodegeneration in AD, while also contributing to insulin resistance and dysfunction of β-cells in diabetes. A previous study showed that PIP4K2A and PIP4K2B play a critical role in mediating the fusion between autophagosomes and lysosomes, by converting PI(5)P to PI(4,5)P2 [34]. The loss of PI5P4Kα and PI5P4Kβ leads to the inhibition of autophagy, autophagosome accumulation, and the prevention of autophagosome–lysosome fusion.

On the other hand, our study highlighted the critical involvement of elevated levels of *PIP4K2A* in the negative regulation of autophagosome assembly, observed in both AD and DM patients’ blood samples. Negative regulation of autophagosome assembly refers to the processes or factors that inhibit or suppress the formation and maturation of autophagosomes, the double-membrane vesicles involved in autophagy [50]. These negative regulators can act at various stages of autophagosome biogenesis, including the initiation, elongation, and closure of the autophagosomal membrane. Dysregulation of these negative regulatory mechanisms can lead to aberrant autophagy and contribute to the pathogenesis of various diseases. The overexpression of PI5P4K2α, which reduces PI(5)P signaling by converting it into PI(4,5)P2, was shown to impair autophagy and associated with the negative regulation autophagosome assembly [51]. In T2DM, autophagy serves dual roles of providing nutrients during fasting and removing cellular damage, while also contributing to pancreatic beta cell dysfunction and insulin resistance [52]. However, under nutrient-rich conditions, the mammalian target of rapamycin complex 1, a critical negative regulator of autophagy, suppresses Unc-51-like kinase 1 which is involved in the initiation of autophagy and autophagosome formation, thereby contributing to the negative regulation of autophagosome assembly [53]. Moreover, a proteomics analysis of the human AD brain demonstrated that among the proteins present in the pTau interactome, PIP4K2α was the only protein exhibiting specificity to oligodendrocytes [54]. This observation is consistent with our findings. In the study conducted by Aber et al., it was observed that oligodendrocytes play a critical role in regulating the turnover of myelin through the coordination of autophagy and endocytosis [55]. The absence of autophagy in oligodendrocytes resulted in abnormalities in myelin, behavioral impairments, glial and neurodegeneration, and ultimately, mortality. The alterations in macro autophagy processes may contribute to circuitry dysfunction observed in neurodegenerative disorders like AD, even in the absence of neuronal loss [55,56].

The dysregulation of cytosolic transport and vesicle-mediated transport processes was implicated in the accumulation of Aβ plaques, a key pathological hallmark of the disease [15,57,58]. PIP4K2α, as a kinase involved in phosphoinositide metabolism and membrane dynamics, may play a role in modulating these transport mechanisms. Under the pathological conditions of AD, oligodendrocytes are particularly susceptible, leading to the breakdown of myelin and the subsequent loss of the protective myelin sheath [59]. Axonal swellings positive for APP are a prominent feature of myelin dysfunction, and impaired axonal transport may enhance Aβ production through increased beta-secretase and APP interactions within axonal transported vesicles [60]. In the context of T2DM, disruptions in cytosolic transport and vesicle-mediated transport were implicated in the dysfunction of insulin secretion and glucose homeostasis [61]. Insulin regulates glucose homeostasis by facilitating glucose uptake into skeletal and fat cells from the bloodstream. This process involves the translocation of glucose transporter 4 to the plasma membrane through fusion with the storage vesicles. Impairment in this process contributes to the early development of insulin resistance and T2DM. PIP4K2α’s involvement in these transport processes suggests its potential role in regulating the trafficking of insulin granules or components critical for insulin exocytosis.

**Figure 8:**
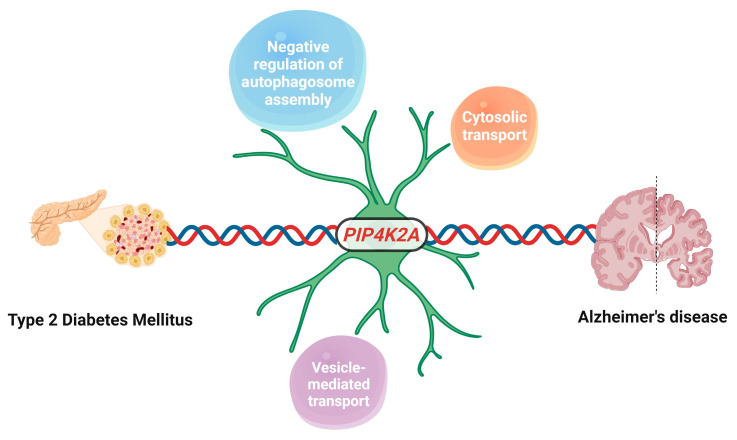
A schematic summary illustrating the potential involvement of *PIP4K2A* in AD and its possible connection to T2DM, predominantly in oligodendrocyte cell type. This illustration was designed via Biorender (https://app.biorender.com/ accessed on 30 January 2024).

In conclusion, our research suggests a potential role for *PIP4K2A* in cellular processes related to AD and its possible connection to T2DM (Figure 8). By exploring the involvement of *PIP4K2A* in oligodendrocytes, we provided insights into its potential contribution to disease pathogenesis through a targeted cell-type approach. While PIP4K2A expression is not exclusive to oligodendrocytes, their significant expression in these cells warrants a focused investigation to unravel the detailed mechanisms by which PIP4K2A contributes to the pathogenesis of AD. In addition, the elevated levels of PIP4K2A in the serum of patients suggest its relevance not only in AD but also in the context of T2DM. This dual association positions PIP4K2A as a candidate biomarker that may reflect the shared pathophysiological processes between these two diseases. Our findings also underscore *PIP4K2A*’s possible involvement in various cellular processes, including vesicle-mediated transport, cytosolic transport, and negative regulation of autophagosome assembly. Besides that, this study also has certain limitations. Firstly, the analysis would benefit from a broader dataset that includes samples from AD patients with T2DM, which could strengthen our conclusions. In addition, the limited number of human serum samples in our current study has restricted our capacity to validate our findings comprehensively. In future research, we aim to not only corroborate these findings but also to validate the mRNA expression levels of PIP4K2A within a more extensive cohort. Moreover, further experimental validation is needed to confirm the change of *PIP4K2A* expression on the progress of AD and T2DM in in vivo and in vitro models. Given that the precise mechanisms through which *PIP4K2A* involves autophagosome assembly, cytosolic transport, and vesicle-mediated transport in AD and T2DM are not yet fully understood, additional studies are required to uncover the molecular pathways involved.

## 4. Materials and Methods

### 4.1. Data Collection

The data utilized in this study were sourced from the GEO database (https://www.ncbi.nlm.nih.gov/geo/ accessed on 30 January 2024). For the AD analysis, we obtained the GSE97760 [62] microarray dataset, which includes gene expression profiles from blood samples of 9 AD patients and 10 control subjects. Gene expression data for T2DM were obtained from the GSE95849 dataset [63], which included 5 samples from individuals with T2DM and 6 samples from healthy controls. Furthermore, three distinct AD tissue datasets were employed for validation, originating from different brain regions: superior frontal gyrus (GSE48350) [64], entorhinal cortex, and hippocampus (GSE5281) [65].

We also evaluated the expression patterns of our hub genes at the single-cell level using high-throughput single-nucleus RNA sequencing datasets obtained from consistent brain regions. In particular, we analyzed datasets from the superior frontal gyrus, entorhinal cortex (GSE147528) [66], and the hippocampus (GSE175814) [67].

The definition and characterization of patient samples are described in Supplementary Table S1.

### 4.2. Data Preprocessing

Prior to conducting further analyses, data preprocessing steps were performed to ensure the quality and reliability of the gene expression data. We utilized the principal component analysis (PCA) to assess the distribution of samples in the multidimensional gene expression space and identify potential outliers. For the diabetes dataset, the sample “GSM2527027” was identified as an outlier based on the PCA analysis and was subsequently removed from further analysis. In contrast, no outliers were detected in the AD dataset, indicating that all samples were suitable for downstream analysis.

### 4.3. Differential Gene Expression Analysis

The DGE analysis was performed using the limma (version 3.58.1) package to compare gene expression profiles in AD patients and control individuals, as well as in individuals with T2DM and control subjects [68]. Genes were considered as DEGs if they exhibited *p*-value < 0.05 and |logFC| > 0.5.

### 4.4. Identification of Commonly Dysregulated Genes in AD and T2DM and The Relevant Signaling Pathways

To identify common genes that exhibit coordinated dysregulation in both diseases, we overlapped the gene lists derived from the DEG analysis in both AD and T2DM. We further investigated underlying potential pathways associated with the shared genes mentioned above. To gain a comprehensive understanding of the biological processes and pathways enriched in the shared genes, we performed an enrichment analysis using clusterProfiler R (version 4.10.1) package [69], in combination with Enrichr database (accessed on 10 December 2023) [70].

### 4.5. Protein–Protein Interaction Network Analysis

To gain insights into the molecular interactions and identify key players in the shared genes between AD and T2DM, we constructed a protein–protein interaction (PPI) network. The PPI network represents the physical interactions between proteins and can provide valuable information about functional relationships and potential hub genes. We utilized publicly available STRING database, which is implement in Network Analyst tool (https://www.networkanalyst.ca/; accessed on 24 December 2023) [71]. These databases integrate various sources of experimental data and predicted interactions to provide a comprehensive view of protein interactions.

### 4.6. AUC-Based Validation and Predictive Modeling of the Common Hub Genes

We utilized gene expression data from the sample of AD and T2DM patients, as well as control individuals and selected the DEGs identified through analysis as features for the machine learning model. Then, the dataset was split into subsets of gene expression matrices, with columns representing gene symbols from the DEGs list and rows corresponding to sample IDs. The DEG expression profiles were used as input variables, while the disease status (AD or T2DM) served as the target variable. Once trained, the model’s performance in predicting the incidence of comorbidity was assessed. We calculated the receiver operating characteristic curve, plotting the true positive rate against the false positive rate, and determined the AUC as a measure of the model’s discriminatory power using the pROC (version 1.18.5) R package [72]. Initially, we applied our selected methods to analyze the AD and DM blood datasets. To further validate the sensitivity and specificity of the chosen genes, we also combined the microarray datasets of brain tissue samples from three distinct regions: the superior frontal gyrus (GSE48350), entorhinal cortex, and hippocampus (both from GSE5281). Genes with an AUC score ≥ 0.7 in all datasets were selected for further analysis.

### 4.7. Single-Cell RNA Sequencing Analysis

We further investigated the expression patterns of our identified genes in the context of AD using single-cell resolution data from various AD studies conducted in different brain regions that we mentioned in the data collection section. The single-cell data were preprocessed following the described methods in the original studies, including filtering and quality control, data normalization, dimensionality reduction, clustering, and t-distributed Stochastic Neighbor Embedding (t-SNE) method for visualization. The cell markers and cell type annotations were validated by The Alzheimer’s Cell Atlas (https://taca.lerner.ccf.org/; accessed on 28 December 2023) [73], SCAD-Brain (https://www.bioinform.cn/SCAD/; accessed on 28 December 2023) [74]. All hub genes were screened in three single-cell datasets obtained from entorhinal cortex, superior frontal cortex (GSE147528), and hippocampus (GSE175814).

### 4.8. Analysis of Key Biological Pathways Related to The Target Gene

We identified the pathways in which our target gene is involved by utilizing several databases, including Gene Ontology and KEGG. To further investigate the functional relevance of these pathways in disease progression, we performed a Gene Set Variation Analysis, which allowed us to compare the pathway signature scores between the disease groups (AD or T2DM) and the control group [75].

### 4.9. Human Subjects for Validation Experiment

A case–control study was conducted using serum samples from Taipei Medical University (TMU) Joint Biobank, comprising four groups: HC, HG, AD and AD–HG. The AD diagnosis adhered to the guidelines of the Diagnostic and Statistical Manual of Mental Disorders (DSM IV) and followed the standards set by the National Institute of Neurological Disorders and Stroke, along with the Alzheimer’s Disease and Related Disorders Association [5,76]. Hyperglycemia was defined as fasting glucose levels exceeding 126 mg/dL (7 mmol/L) and glycohemoglobin A1 (HbA1c) levels exceeding 6.5%. The research protocol was approved by the TMU Joint Institutional Review Board (JIRB No: N202203066).

### 4.10. Western Blotting

The total protein concentration of each serum sample was measured using a Protein Assay kit from Bio-Rad Laboratories, Inc. in Hercules, CA, USA. Following this, one microliter of serum was diluted 10 times with 1X phosphate-buffered saline. The diluted protein samples were mixed with loading buffer and then denatured at 95 °C for five minutes.

The prepared protein samples were separated using 10% SDS-PAGE and transferred onto polyvinylidene fluoride membrane. After blocking and washing, the membrane was incubated with a monoclonal primary antibody specifically against PIP4K2A (Cell Signaling Technology, #5527, Danvers, MA, USA) at 4 °C overnight. After thorough washing, the membrane was incubated with HRP-labeled goat-anti-rabbit IgG secondary antibodies (GeneTex, GTX213110-01, Irvine, CA, USA) at 25 °C for 1 h. Subsequently, the membrane was washed and incubated in a chemiluminescent substrate from Bio-Rad. Bands were detected and imaged using the ImageQuant LAS 4000 system. Band densities were analyzed and quantified using ImageJ software (version 1.53t).

### 4.11. Statistical Analysis

All statistical analyses were performed using R software (version 4.1.3). Prism software (version 9.0.2) was used for statistical analysis and graphical representations of the experimental data.

## Figures and Tables

**Table 1:** The AUC score of eight candidate genes in AD and DM microarray datasets.

Gene	DM—Blood	AD—Blood	AD—Entorhinal Cortex	AD—Superior Frontal Gyrus	AD—Hippocampus
*BAZ1A*	0.97	0.88	0.82	0.89	0.95
*FGD4*	0.83	0.89	0.87	0.88	0.91
*NOTCH2NL*	0.90	0.98	0.90	0.82	0.82
*PIP4K2A*	0.90	0.89	0.93	0.71	0.78
*SNAP23*	0.93	0.96	0.83	0.89	0.76
*ZFP36L1*	0.93	0.96	0.85	0.84	0.80
*ELAVL4*	0.87	0.73	0.89	0.79	0.83
*MAP7D2*	0.90	0.92	0.74	0.71	0.88

## Data Availability

All the data analyzed in this study are available through the GEO database, which can be assessed at: https://www.ncbi.nlm.nih.gov/geo/ accessed on 30 January 2024.

## Supplementary Materials

The following supporting information can be downloaded at: https://www.mdpi.com/article/10.3390/ijms25126640/s1.

## Abbreviations

AD, Alzheimer’s disease; Aβ, amyloid-beta; APP, amyloid precursor protein; AUC, area under the curve; DEG, differentially expressed gene, DGE, differential gene expression; GEO, gene expression omnibus; KEGG, Kyoto Encyclopedia of Genes and Genome; PIP4K2A, phosphatidylinositol-5-phosphate 4-kinase type 2 alpha; ROC, receiver operating characteristic; T2DM, type 2 diabetes mellitus; t-SNE, t-distributed Stochastic Neighbor Embedding.
