# Supplementary material for: Exploring the Potential Role of Oligodendrocyte-Associated PIP4K2A in Alzheimer’s Disease Complicated with Type 2 Diabetes Mellitus via Multi-Omic Analysis
Source: Int J Mol Sci. 2024 Jun 17;25(12):6640. doi: 10.3390/ijms25126640 (PMC11204139; doi:10.3390/ijms25126640)
Supplement: Supplementary file 1 [file ijms-25-06640-s001.zip › ijms-3039573-supplementary.pdf]

SUPPLEMENTARY MATERIALS

Table of contents

Supplementary Figure S1. Characteristics of GSE95849 (T2DM dataset) before and after PCA. 2

Supplementary Figure S2. The volcano plot revealed differentially expressed genes in the comparison of oligodendrocyte and other cell types. 3

Supplementary Figure S3. Immune infiltration analysis of *PIP4K2A* in AD and T2DM blood datasets. 4

Supplementary Table S1. Definition and characterization of patient samples 5

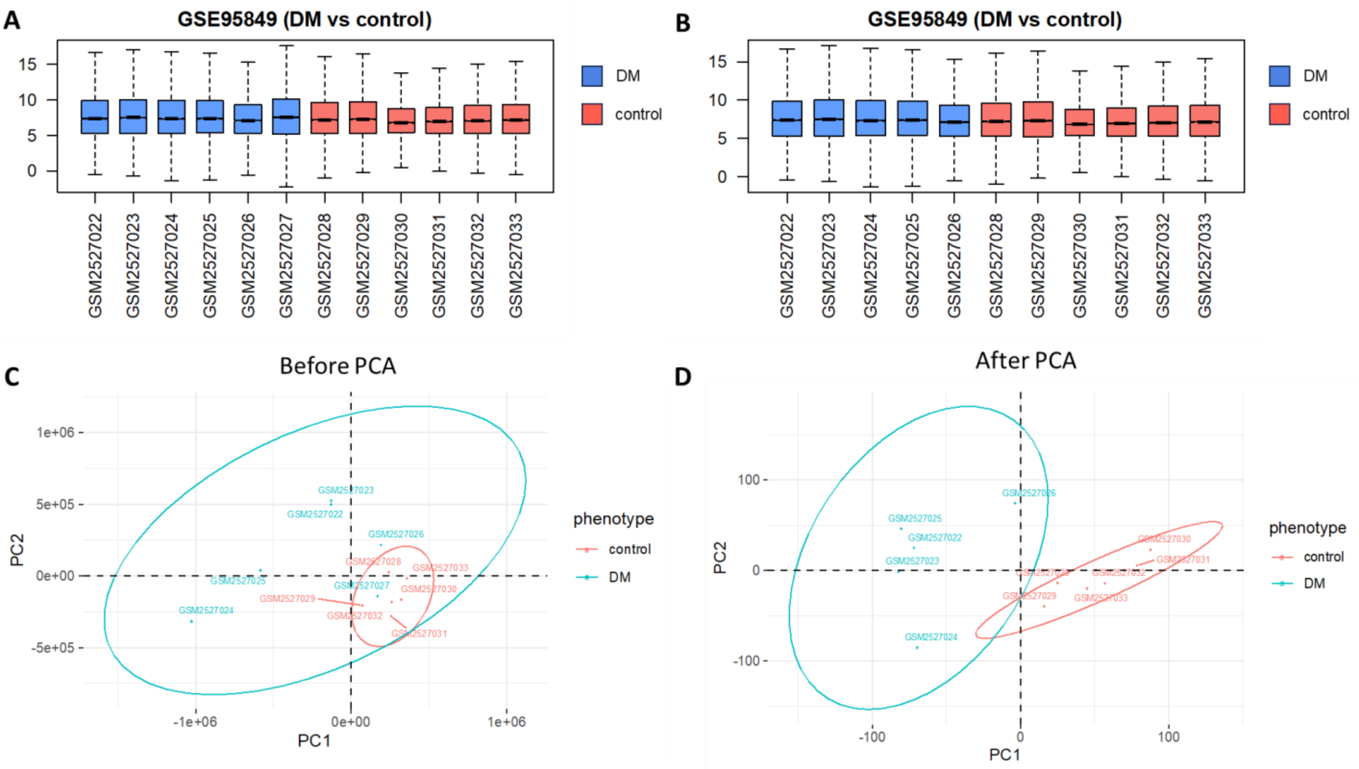

Supplementary Figure S1. Characteristics of GSE95849 (T2DM dataset) before and after PCA. (A-B) Box plots illustrate the sample distributions and (C-D) illustrate the PCA maps of GSE95849 dataset before and after excluding outlier, respectively.

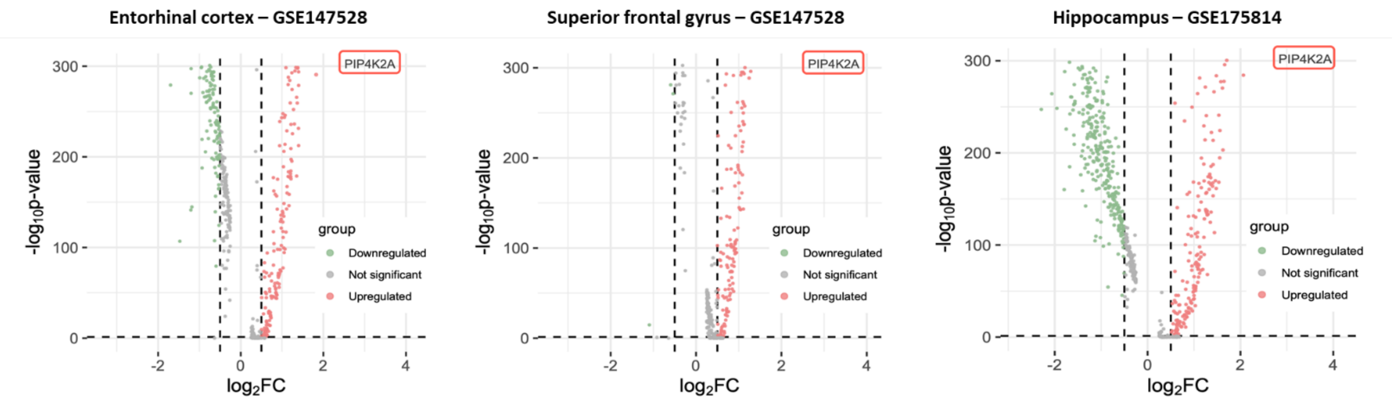

Supplementary Figure S2. The volcano plot revealed differentially expressed genes in the comparison of oligodendrocyte and other cell types.

PIP4K2A exhibited significantly higher expression levels in oligodendrocytes across all three single-cell datasets from distinct brain regions entorhinal cortex, superior frontal gyrus, and hippocampus.

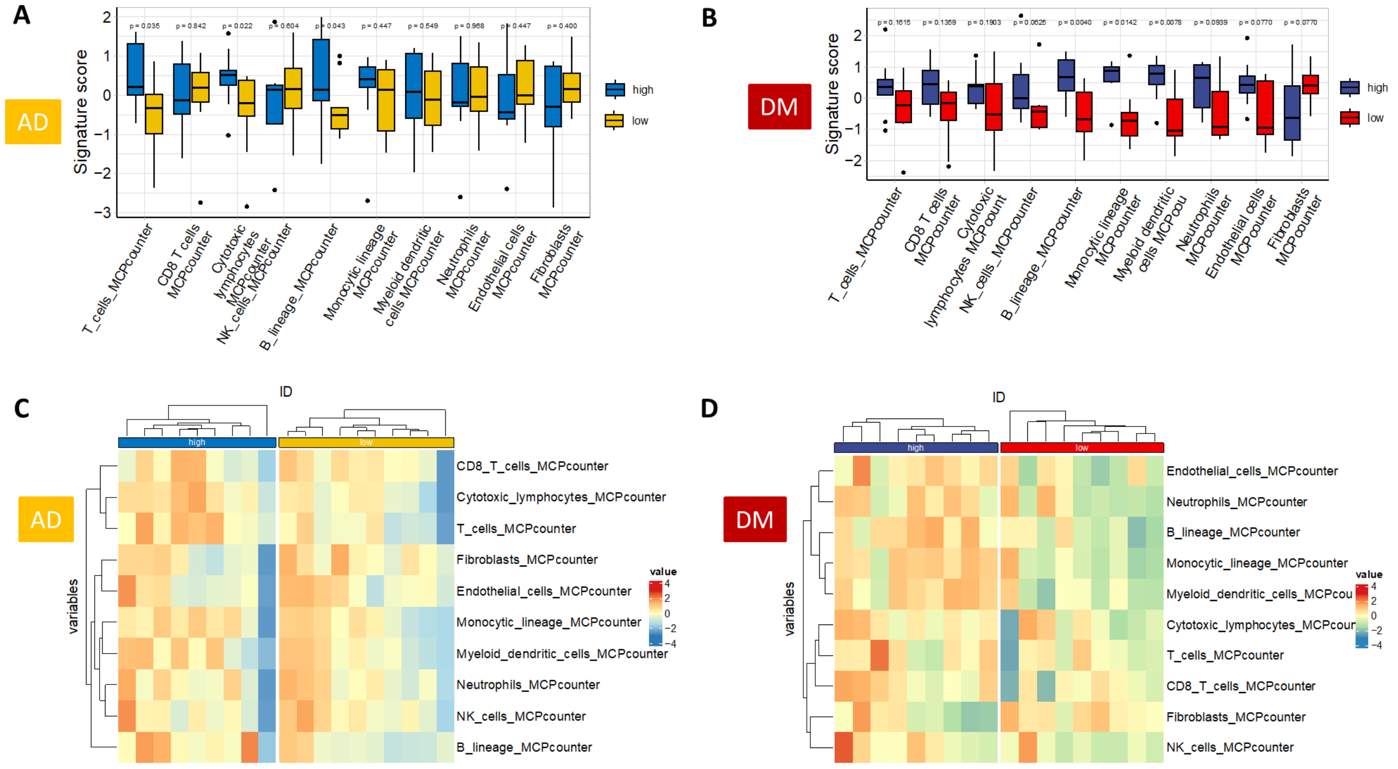

Supplementary Figure S3. Immune infiltration analysis of *PIP4K2A* in AD and T2DM blood datasets.

(A-B) Varied levels of MCPcounter immune signature observed between high and low *PIP4K2A* expression. **(C-D)** Heatmaps illustrating significant alterations in immune scores in AD and T2DM samples based on *PIP4K2A* expression levels.

Supplementary Table S1. Definition and characterization of patient samples.

Dataset	Conditions and sample sources	Dataset type	Sample size	Ethnicity	Sex	Age	Disease stage
GSE95849	DM (Blood)	Microarray	DM: 6	NA	Female	53.83 ± 10.80	NA
			Control: 6	NA	Female	51.17 ± 6.08	NA
GSE97760	AD (Blood)	Microarray	AD: 9	Caucasian: 7<br>African American: 2	Female	79.3 ± 12.3	NA
			Control: 10	Caucasian: 10	Female	72.1 ± 13.1	NA
GSE5281	AD (Entorhinal Cortex)	Microarray	AD: 10	Unknown	Female: 6<br>Male: 4	84.8 ± 6.2<br>86.75 ± 7.27	NA
			Control: 13	Caucasian	Female: 3<br>Male: 10	90.67 ± 10.2<br>77.2 ± 6.56	NA
	AD (Hippocampus)		AD: 10	Caucasian: 1<br>Unknown: 9	Female: 4<br>Male: 6	76.25 ± 6.5<br>78.83 ± 5.49	NA
			Control: 13	Caucasian	Female: 3<br>Male: 10	87.67 ± 14.5<br>77.2 ± 6.56	NA
GSE48350	AD (Superior frontal gyrus)	Microarray	AD: 21	NA	Female: 14<br>Male: 7	86.8 ± 6.13<br>87.7 ± 6.7	NA
			Control: 48	NA	Female: 24<br>Male: 24	64.2 ± 23.6<br>55.7 ± 26.2	NA
GSE147528	AD (Superior frontal gyrus)<br>AD (Entorhinal Cortex)	scRNA-seq	Braak 0: 3<br>Braak 2: 4<br>Braak 6: 3	NA	Male: 10	60.3 ± 8.57<br>81.75 ± 7.59<br>78.6 ± 4.7	Braak 0<br>Braak 2<br>Braak 6
GSE175814	AD (Hippocampus)	scRNA-seq	Braak 0: 2<br>Braak 3/4: 2	NA	Male: 4	Braak 0: 66 (mean)<br>braak 3/4: 69.5 (mean)	Braak 0<br>Braak 3/4
In house dataset	AD & Hyperglycemia (serum)	Western blot	Healthy control: 3	Asian	Female: 2<br>Male: 1	74.6 ± 5.2	NA
			Hyperglycemia: 3		Female: 2<br>Male: 1	72.6 ± 1.7	NA
			AD: 3		Female: 2<br>Male: 1	79.6 ± 2.1	NA
			AD with hyperglycemia: 3		Female: 2<br>Male: 1	78.6 ± 4.1	NA
